# The effectiveness of a provincial symptom assessment program in reaching adolescents and young adults with cancer: A population‐based cohort study

**DOI:** 10.1002/cam4.4401

**Published:** 2021-11-05

**Authors:** Sumit Gupta, Rinku Sutradhar, Qing Li, Natalie Coburn

**Affiliations:** ^1^ Division of Haematology/Oncology The Hospital for Sick Children Toronto Canada; ^2^ Cancer Research Program ICES Toronto Canada; ^3^ Institute for Health Policy, Evaluation and Management University of Toronto Toronto Canada; ^4^ Faculty of Medicine University of Toronto Toronto Canada; ^5^ Dalla Lana School of Public Health University of Toronto Toronto Canada; ^6^ Department of Surgery Sunnybrook Health Sciences Centre Toronto Canada; ^7^ Department of Surgery University of Toronto Toronto Canada; ^8^ Sunnybrook Research Institute Toronto Canada

**Keywords:** adolescents, cancer, patient‐reported outcomes, population‐based, symptoms, young adults

## Abstract

**Background:**

Symptom control is prioritized by cancer patients and may improve overall survival. Ontario, Canada thus offers all cancer patients screening using the Edmonton Symptom Assessment System (ESAS) at outpatient cancer‐related visits. We determined whether this initiative reached adolescents and young adults (AYA) and factors associated with screening in this population.

**Methods:**

We linked all Ontario AYA diagnosed with cancer 2010–2018 aged 15–29 years to population‐based databases identifying outpatient visits and ESAS screening. For each 2‐week period in the year post‐diagnosis, AYA with cancer‐related visits were categorized as “unscreened” (no ESAS score) versus “screened” (≥1 ESAS score). Demographic and disease‐related covariates were examined.

**Results:**

Among 5435 AYA, 4204 (77.4%) had ≥1 ESAS screen. Within any 2‐week period, only 30%–44% of AYA attending cancer‐related visits were screened. Patients with hematologic malignancies were least likely to be screened [odds ratio (OR) vs. breast cancer 0.77, 95% confidence interval (95% CI) 0.67–0.88; *p* < 0.001]. AYA in remote Northern or rural areas had equivalent or higher rates of ESAS screening compared to those in high‐income urban areas. However, AYA living in the lowest income urban neighborhoods were less likely to be screened (OR 0.86, 95% CI 0.77–0.97; *p* = 0.01).

**Conclusions:**

Within a population‐wide symptom assessment program, while AYA living in rural and remote areas had high rates of screening, than those in low‐income urban areas were substantially less likely to be screened. Though patients with hematologic cancers suffer from particularly high symptom burdens, they were also less likely to be screened. Interventions targeting AYA are required to increase uptake.

## Graphical abstract

Symptom control is prioritized by cancer patients; we assessed whether a population‐based symptom screening program was effective in screening adolescents and young adults (AYA). AYA living in rural and remote areas had high rates of screening, but those living in low‐income urban areas and those with hematologic cancers had lower screening rates. AYA‐specific measures may be warranted.

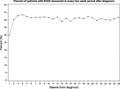

## 1 INTRODUCTION

Across all cancer types and throughout the cancer journey, patients experience significant symptom burdens as a result of both the cancer and its treatment.^1^, ^2^ Suboptimal symptom control is common, impacting on patients’ quality of life.^3^, ^4^, ^5^, ^6^ Though traditionally ascertained by healthcare providers, systematic use of patient‐reported outcome measures (PROMs) with interventions to support patients who report high scores have been shown to improve quality of life and clinical outcomes, including in recent landmark randomized control trials.^7^, ^8^, ^9^, ^10^, ^11^ This has led some institutions and jurisdictions to incorporate PROMs to assess symptom burden in cancer patients at regular intervals.

Adolescents and young adults (AYA) are a vulnerable subpopulation of cancer patients, in whom outcome disparities have been noted.^12^, ^13^, ^14^ These disparities have been partially attributed to healthcare system‐level factors^15^, ^16^; cancer care for AYA is scattered across different settings (adult vs. pediatric, cancer centers vs. community hospitals), none designed with their unique needs in mind. Programs designed primarily for older cancer patients may therefore not reach AYA, or may do so inequitably across socioeconomic status or geography.

Our primary objective was therefore to determine rates of uptake of a universal symptom screening program in Ontario, Canada among AYA with cancer. We also aimed to identify and describe subgroups of AYA patients with lower rates of screening in order to inform future interventions targeting these subgroups.

## 2 METHODS

### 2.1 Study setting

Canadian healthcare is delivered by provincial governments via universal health insurance plans. In Ontario, cancer care delivery is coordinated and managed by Ontario Health‐Cancer Care Ontario (OH‐CCO). Adult cancer care is delivered through both Regional Cancer Centers (RCCs) and community centers.

Edmonton Symptom Assessment System (ESAS) is a valid and reliable PROM that was developed to assess the presence and severity of nine common cancer‐associated symptoms: pain, tiredness, drowsiness, nausea, lack of appetite, shortness of breath, depression, anxiety, and overall well‐being.^17^, ^18^ In the version used in Ontario, each symptom is scored on an 11‐point numeric rating scale that ranges from 0 (no symptoms) to 10 (worst possible symptoms). ESAS scores for each symptom are commonly categorized as follows: no symptoms (0), mild symptoms (1–3), moderate symptoms (4–6), and severe symptoms (7–10).^19^, ^20^

In 2007, OH‐CCO implemented a province‐wide program aiming to screen cancer patients at cancer‐related outpatient visits for common symptoms using ESAS to assist providers in providing supportive care with the goal of achieving symptom control. The ESAS form used by OH‐CCO may be seen in Appendix S1. Originally targeting patients with lung cancer or receiving palliative care at RCCs, this program was then expanded to other cancer sites in subsequent years. Overall, ESAS screening was broadly available at RCCs by 2010.^21^, ^22^, ^23^ Though multiple non‐RCC community centers also use ESAS, implementation among non‐RCCs has been more variable. OH‐CCO maintains a Web‐based platform which is used to administer ESAS in cancer clinics with patients, using kiosks, computer tablets, or manual input of paper forms. The questionnaire has been translated into multiple languages. Inpatients are not screened with ESAS. All symptom scores are collected centrally in the Symptom Management Reporting Database (SMRD). Additional details have been previously published.^21^, ^22^, ^23^

### 2.2 Study design and population

We conducted a retrospective population‐based cohort study. All AYA diagnosed with primary cancer aged 15–29 years at diagnosis between 1 January 2010 and 30 June 2018 were identified using the Ontario Cancer Registry. Patients diagnosed and treated in pediatric institutions, who do not use ESAS, were excluded.

The Activity Level Reporting (ALR) database contains data elements pertaining to patient‐level activity within the cancer system, mainly focused on radiation and systemic therapies, as well as outpatient oncology clinic visits. Cancer centers are mandated to report patient‐level cancer activity to the ALR. Patients without any ALR records in the first year of diagnosis were excluded, as these mainly represent patients who did not receive any service at either an RCC or a community cancer center (e.g., patients with surgically resected thyroid cancer, removal of colonic polyps). Given different patterns of ESAS uptake in non‐RCCs as noted above, patients whose ALR‐identified visits occurred exclusively at non‐RCCs were also excluded, though patients whose visits occurred at both RCCs and non‐RCCs were retained. Patients were linked deterministically to population‐based health services databases housed at ICES, a research institute encompassing an array of Ontario health‐related data. These datasets were linked using unique encoded identifiers and analyzed at ICES.

### 2.3 Outcome

Our primary outcome was the receipt of an ESAS screen as identified through the SMRD. The first year after diagnosis was divided into 2‐week periods. For each period, a patient was considered “unscreened” if they had at least one ALR visit but no ESAS screen records, and “screened” if they had at least one ALR visit and at least one ESAS screen record during that time interval. Patients did not contribute to 2‐week time intervals in which they did not have any ALR visits. Patients were followed from diagnosis to the earliest of death, provincial insurance ineligibility for two consecutive quarters, or 1 year.

### 2.4 Covariates

We examined several potential baseline predictors of being ESAS screened. Demographic characteristics included age at diagnosis (continuous) and sex. Neighborhood income quintile and urban/rural status were determined using data from the Canadian census.^24^, ^25^ Regional location was categorized by the five main Ontario health regions (Central, East, North, Toronto, and West). Cancer type was categorized using the following broad categories as identified by the Ontario Cancer Registry: hematologic, melanoma, central nervous system, sarcoma, testicular/ovarian, breast, colorectal, thyroid, and other. Time period of diagnosis was defined as early (2010–2014) and late (2015–2018).

For each 2‐week period, institution type (RCC vs. non‐RCC) was defined by identifying the institution that the patient visited most often in the 30 days prior to the start of the period. Thus, the same patient may have had different institutions assigned to different 2‐week periods. If the patient did not have any ALR visits in the prior 30 days, the first institution visited during the 2‐week period itself was used.

### 2.5 Analyses

The proportion of patients ESAS screened in each 2‐week interval was calculated and depicted graphically. Predictors of ESAS screening were determined using univariate and multivariable logistic regression models. All variables were included in multivariable models regardless of univariate significance. Correlation due to repeated measures from the same individual (taken across multiple 2‐week intervals) was accounted for using a generalized estimating equations approach. We also assessed initial estimates of correlation within institutions, which revealed a near zero intraclass correlation coefficient (ICC = 0.04); clustering at the institutional level was therefore not accounted for. Statistical significance was defined as *p* < 0.05. Analyses were performed using SAS, version 9.4 (SAS Institute). Ethics approval was obtained at The Hospital for Sick Children and Sunnybrook Health Sciences Centre. Informed consent was not required.

## 3 RESULTS

Of the 9399 identified patients, 649 (6.9%) were diagnosed and treated at pediatric institutions. Of the 8750 AYA diagnosed and treated at adult institutions, 2949 (33.7%) had no ALR visit within the first year of diagnosis and were thus excluded. Confirming previous assumptions that such patients represent cancer diagnoses that do not require services at a cancer center, 1416 (48.0%) had thyroid cancer, only 47 (0.02%) died within a year of diagnosis, and only 82 (0.03%) were ESAS screened within a year. Of the remaining 5801 patients with ALR visits within the first year, 366 (6.3%) only had ALR visits at non‐RCCs and thus also excluded. These excluded patients were less likely to live in rural areas (13/366 [3.6%] vs. 535/5435 [9.8%]; *p* < 0.001] and more likely to be diagnosed in the later time period (242/366 [66.1%] vs. 2175/5435 [40.0%]; *p* < 0.001).

**FIGURE 1:**
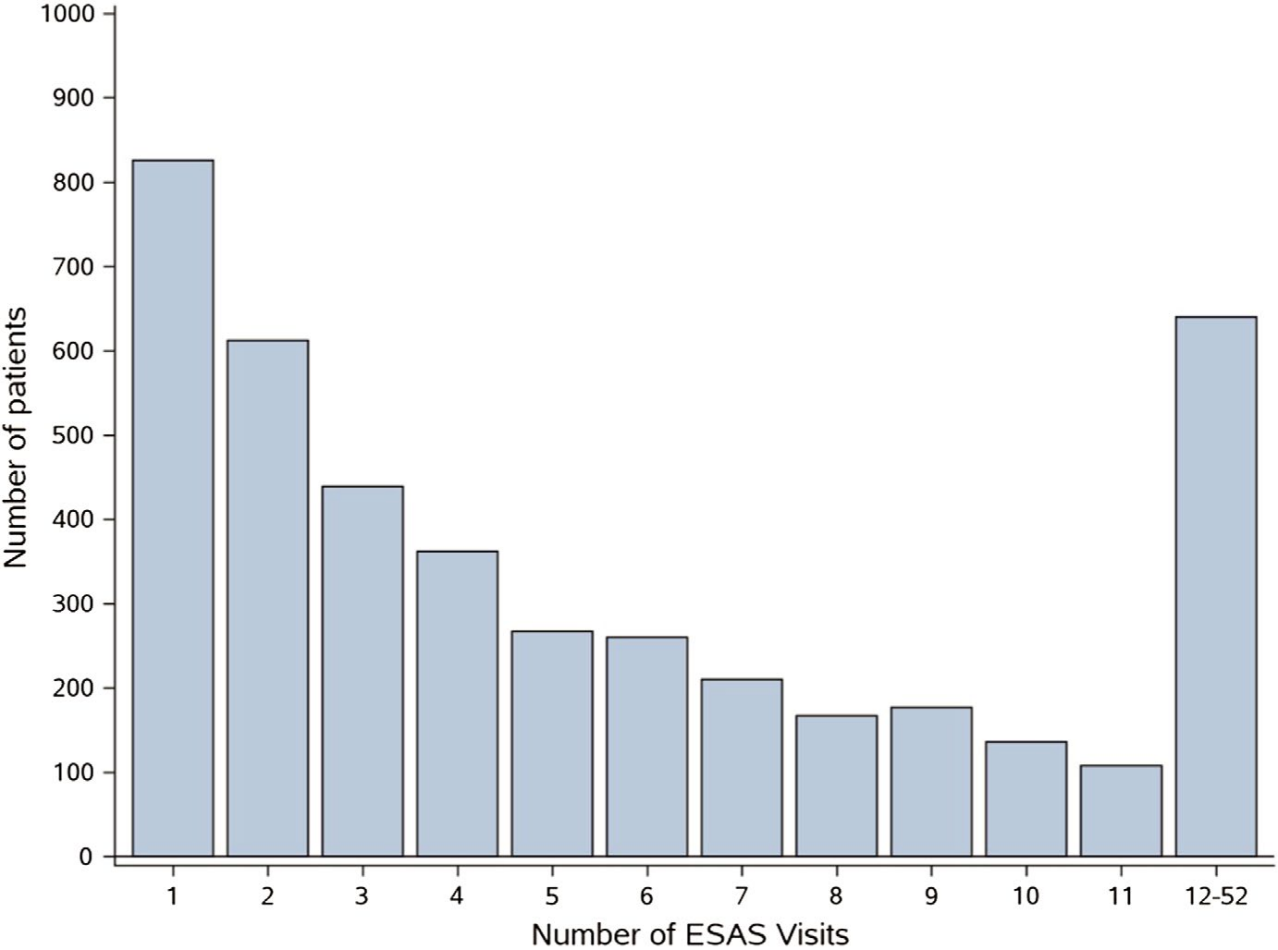
Frequency of ESAS screen‐associated visits within the first year of diagnosis. ESAS, Edmonton Symptom Assessment System

**FIGURE 2:**
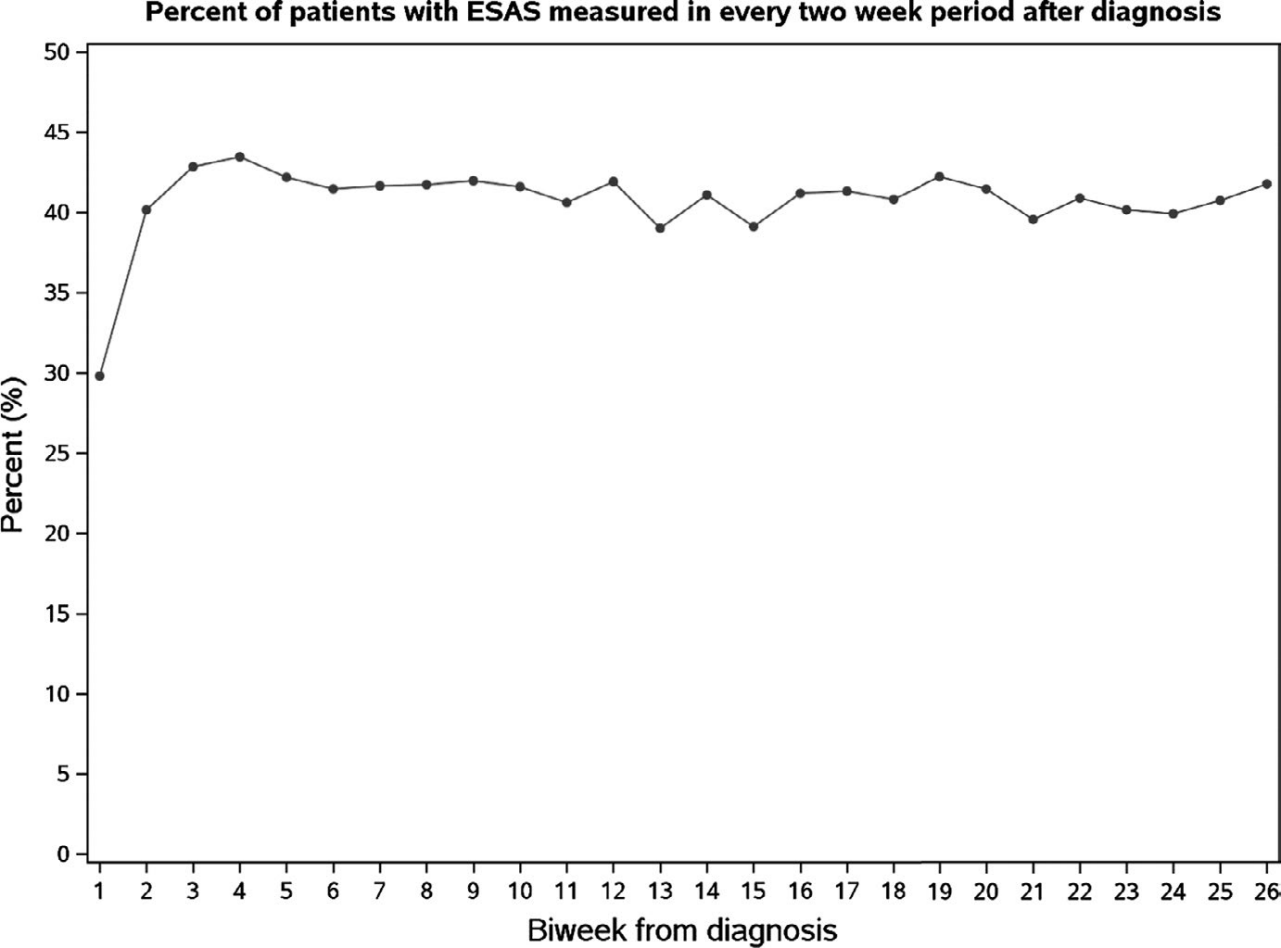
Percentage of adolescents and young adults ESAS screened within each 2‐week interval following from diagnosis. ESAS, Edmonton Symptom Assessment System

The final study cohort therefore comprised of 5435 AYA, whose characteristics are shown in Table 1. Of the cohort, 4204 patients (77.4%) were screened with ESAS at least once during the first year following diagnosis. The median number of ESAS screens in the first year was 4 (interquartile range 2–9; Figure 1). Figure 2 shows the proportion of patients with ALR visits within each 2‐week period who were also ESAS screened. Only 29.8% of patients were screened in the first 2‐week interval of post‐diagnosis, though this figure then rose to approximately 40% for the remaining intervals. When ESAS screens were present, almost all (99.5%) could be considered complete, with scores imputed for all nine ESAS symptoms. Of ESAS screens included in this study, 64.5% were completed electronically in the clinic.

**TABLE 1:** Demographic and disease characteristics of study cohort (*N* = 5435)

	*N* (%)	Median (IQR)
Age (years)		25 (22–27)
Sex		
Male	2809 (51.7)	
Female	2626 (48.3)	
Time period		
Early (2010–2014)	3260 (60.0)	
Late (2015–2018)	2175 (40.0)	
Neighborhood income quintile		
Rural	535 (9.9)	
Urban Q1 (lowest)	924 (17.0)	
Urban Q2	1026 (18.9)	
Urban Q3	952 (17.6)	
Urban Q4	975 (18.0)	
Urban Q5 (highest)	1009 (18.6)	
Cancer type		
Hematologic	1748 (32.2)	
Melanoma	343 (6.3)	
CNS	335 (6.2)	
Sarcoma	245 (4.5)	
Testicular/ovarian	1159 (21.3)	
Breast	361 (6.6)	
Colorectal	197 (3.6)	
Thyroid	331 (6.1)	
Other	716 (13.2)	
Region		
Central	1737 (32.0)	
East	1268 (23.3)	
North	350 (6.4)	
Toronto	585 (10.8)	
West	1493 (27.5)	

Note

N: number.

Abbreviations: CNS, central nervous system; IQR, interquartile range.

Table 2 shows the predictors of receiving an ESAS screen. AYA with hematologic cancers were significantly less likely to be screened (odds ratio [OR] vs. breast cancer 0.77, 95% confidence interval [95% CI] 0.67–0.88; *p* = 0.0002) as were those at non‐RCC centers (OR 0.48, 95% CI 0.42–0.55; *p* < 0.0001). No differences between rural AYA and those living in high‐income urban neighborhoods were seen. AYA living in the Northern region were in fact substantially more likely to be ESAS screened as compared to those in Toronto (OR 1.64, 95% CI 1.38–1.93; *p* < 0.0001). Among urban AYA however, those in the lowest two neighborhood income quintiles were less likely to be ESAS screened as opposed to those residing in the highest income quintile.

**TABLE 2:** Univariate and multivariable predictors of being ESAS screened

	Univariate		Multivariable	
	OR (95% CI)	*p* value	OR (95% CI)	*p* value
Age (per 10 years)	1.07 (0.97–1.18)	0.18	1.05 (0.95–1.16)	0.37
Sex				
Male	Ref	Ref	Ref	Ref
Female	1.04 (0.97–1.11)	0.31	1.08 (1.00–1.17)	0.05
Time period				
Early (2010–2014)	Ref	Ref	Ref	Ref
Late (2015–2018)	**1.32 (1.23–1.42)**	**<0.0001**	**1.38 (1.29–1.48)**	**<0.0001**
Neighborhood income quintile				
Rural	1.05 (0.92–1.20)	0.47	0.93 (0.81–1.07)	0.34
Urban Q1 (lowest)	**0.88 (0.79**–**0.99)**	**0.04**	**0.86 (0.77–0.97)**	**0.01**
Urban Q2	0.91 (0.81–1.03)	0.13	**0.88 (0.79–0.99)**	**0.03**
Urban Q3	0.96 (0.86–1.08)	0.50	0.96 (0.85–1.07)	0.44
Urban Q4	0.94 (0.84–1.05)	0.28	0.94 (0.84–1.06)	0.32
Urban Q5 (highest)	Ref	Ref	Ref	Ref
Cancer type				
Hematologic	**0.79 (0.69–0.89)**	**0.0003**	**0.77 (0.67–0.88)**	**0.0002**
Melanoma	0.99 (0.82–1.20)	0.94	0.93 (0.77–1.12)	0.45
CNS	0.95 (0.79–1.14)	0.57	0.90 (0.75–1.07)	0.24
Sarcoma	0.96 (0.80–1.15)	0.63	0.93 (0.77–1.13)	0.49
Testicular/ovarian	1.08 (0.94–1.23)	0.29	1.05 (0.91–1.22)	0.51
Breast	Ref	Ref	Ref	Ref
Colorectal	0.95 (0.76–1.18)	0.62	0.92 (0.74–1.14)	0.44
Thyroid	0.83 (0.68–1.02)	0.08	0.82 (0.67–1.00)	0.05
Other	1.04 (0.90–1.21)	0.60	0.99 (0.85–1.15)	0.89
Region				
Central	1.12 (0.99–1.27)	0.07	**1.16 (1.02–1.31)**	**0.02**
East	1.04 (0.91–1.18)	0.60	1.08 (0.94–1.23)	0.28
North	**1.53 (1.30–1.80)**	**<0.0001**	**1.64 (1.38–1.93)**	**<0.0001**
Toronto	Ref	Ref	Ref	Ref
West	**1.63 (1.44–1.85)**	**<0.0001**	**1.65 (1.46–1.88)**	**<0.0001**
Institution type				
RCC	Ref	Ref	Ref	Ref
Non‐RCC	**0.49 (0.43–0.56)**	**<0.0001**	**0.48 (0.42–0.55)**	**<0.0001**

Note

Bolded values indicate *p*‐values of < 0.05.

Abbreviations: CI, confidence interval; CNS, central nervous system; OR, odds ratio; RCC, regional cancer center.

**FIGURE 3:**
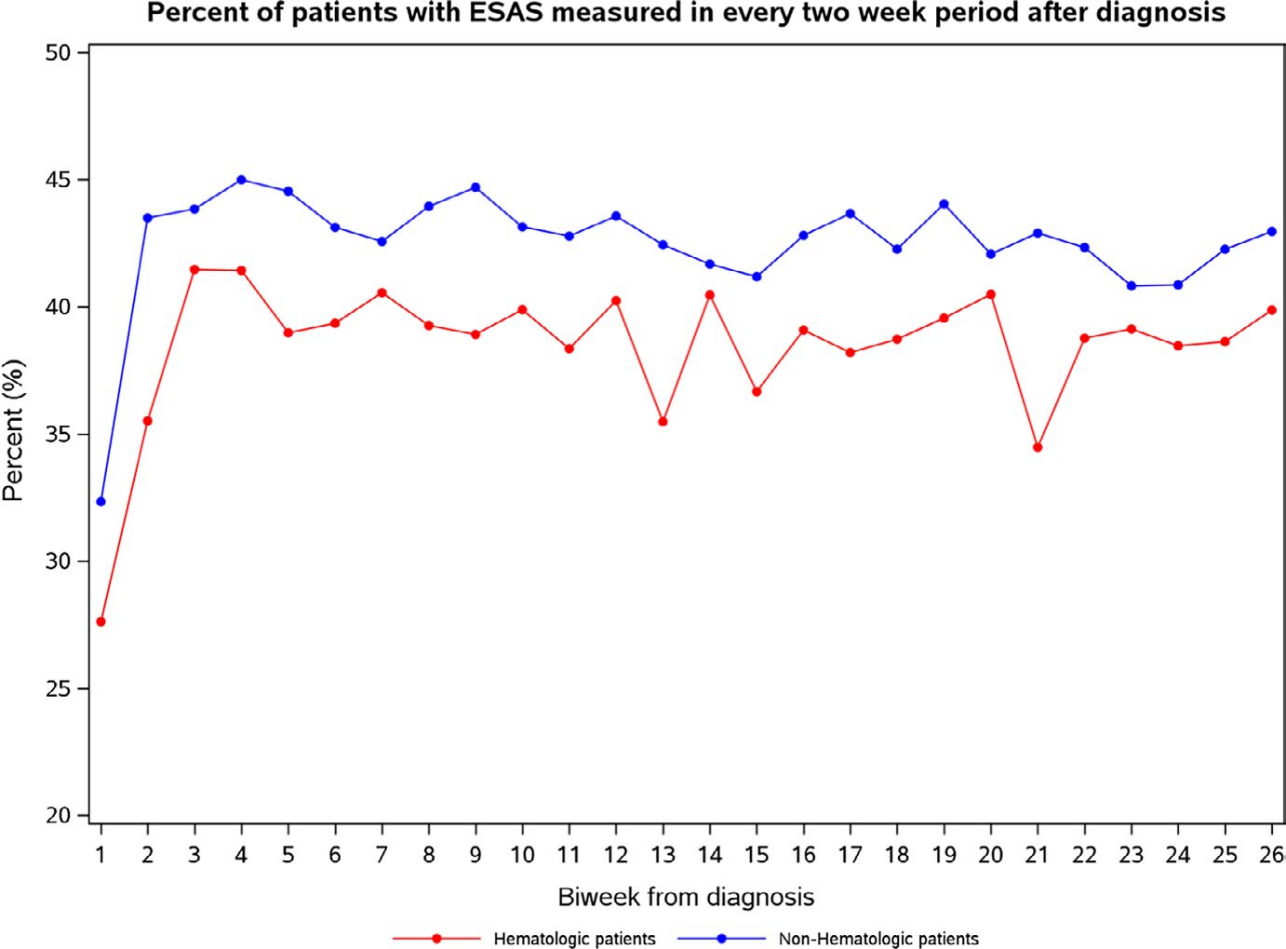
Percentage of adolescents and young adults ESAS screened within each 2‐week interval following from diagnosis, stratified by hematologic versus nonhematologic cancer. ESAS, Edmonton Symptom Assessment System

Given the lower rates of ESAS screening among hematologic patients (Figure 3), we conducted a sensitivity analysis determining predictors of ESAS among AYA with hematologic cancers only (Table 3). For the most part, predictors of ESAS screening were similar to those within the whole AYA cohort, with those in the lowest neighborhood income quintile, those residing in Toronto, and those treated at non‐RCC centers were statistically less likely to be screened. However, compared to AYA with Hodgkin lymphoma, both those with non‐Hodgkin lymphoma (OR 0.81, 95% CI 0.70–0.94; *p* = 0.004) and those with leukemia (OR 0.62, 95% CI 0.53–0.73; *p* < 0.001) were less likely to be screened.

**TABLE 3:** Univariate and multivariable predictors of being ESAS screened, restricted to AYA with hematologic cancers

	Univariate		Multivariable	
	OR (95% CI)	*p* value	OR (95% CI)	*p* value
Age (per 10 years)	0.90 (0.76–1.07)	0.25	0.94 (0.80–1.12)	0.52
Sex				
Male	Ref	Ref	Ref	Ref
Female	0.99 (0.87–1.12)	0.84	0.98 (0.87–1.11)	0.79
Time period				
Early (2010–2014)	Ref	Ref	Ref	Ref
Late (2015–2018)	**1.29 (1.14–1.45)**	**<0.001**	**1.33 (1.18–1.50)**	**<0.001**
Neighborhood income quintile				
Rural	1.04 (0.81–1.32)	0.78	0.96 (0.75–1.23)	0.77
Urban Q1 (lowest)	**0.76 (0.62–0.92)**	**0.006**	**0.79 (0.65–0.96)**	**0.02**
Urban Q2	0.89 (0.73–1.09)	0.27	0.87 (0.71–1.07)	0.18
Urban Q3	0.87 (0.71–1.07)	0.19	0.86 (0.70–1.06)	0.16
Urban Q4	0.94 (0.78–1.15)	0.55	0.93 (0.76–1.13)	0.45
Urban Q5 (highest)	Ref	Ref	Ref	Ref
Cancer type				
Hodgkin lymphoma	Ref	Ref	Ref	Ref
Leukemia	**0.66 (0.56–0.77)**	**<0.001**	**0.62 (0.53–0.73)**	**<0.001**
Non‐Hodgkin lymphoma	**0.80 (0.69–0.92)**	**0.002**	**0.81 (0.70–0.94)**	**0.004**
Region				
Central	1.18 (0.94–1.47)	0.15	1.19 (0.95–1.50)	0.13
East	0.99 (0.78–1.26)	0.94	0.97 (0.76–1.24)	0.80
North	**1.54 (1.16–2.03)**	**0.003**	**1.61 (1.21–2.15)**	**0.001**
Toronto	Ref	Ref	Ref	Ref
West	**1.87 (1.49–2.34)**	**<0.001**	**1.79 (1.43–2.26)**	**<0.001**
Institution type				
RCC	Ref	Ref	Ref	Ref
Non‐RCC	**0.54 (0.44–0.65)**	**<0.001**	**0.50 (0.42–0.61)**	**<0.001**

Note

Bolded values indicate *p*‐values of < 0.05.

Abbreviations: CI, confidence interval; OR, odds ratio; RCC, regional cancer center.

## 4 DISCUSSION

In this large, population‐based cohort of AYA with cancer, we found that approximately 40% of AYA were screened for symptom burdens during any given 2‐week period within the first year following the cancer diagnosis. However, despite the presence of a universal screening program, patients with hematologic malignancies, those living in low‐income urban neighborhoods, and those not attending RCCs were substantially less likely to be screened.

The identification of burdensome symptoms among through PROMs has been shown not only to improve quality of life for cancer patients through adequate symptom control, but also to decrease health care utilization and improve clinical outcomes.^7^, ^8^, ^9^, ^10^, ^11^ In response to this evidence, several jurisdictions, including Ontario, have implemented programs aiming to screen cancer patients for symptoms at regular intervals. Despite the goal of universality of such programs, disparities in uptake have been noted. In Ontario for example, among the general cancer population, geographic and socioeconomic disparities were demonstrated, but had narrowed over time.^26^

Accurate identification and adequate control of cancer and cancer treatment‐related symptoms among AYA may be of particular importance. Though the literature is sparse, authors have postulated that perceptions of symptom experience and severity may differ between AYA and older patients.^27^ Small sample studies have found that AYA with breast and colon cancer were more likely to suffer multiple symptoms as compared to older patients, and that these symptoms were slower to resolve.^28^, ^29^ Common AYA malignancies such as Hodgkin lymphoma and testicular cancer are also associated with high symptom burdens.^30^, ^31^ Given this, it is crucial to know whether universal programs implementing PROMs without specifically targeting AYA are nonetheless successfully reaching this population, both overall and also equitably across subgroups. Previous work had demonstrated that overall in Ontario, younger cancer patients were slightly more likely to undergo ESAS screening than older patients, with a 1% increased chance of being screened for every year decrease in age at the time of diagnosis, but did not specifically examine trends in AYA.^26^ As AYA cancers represent a small portion of the overall cancer burden, risk factors identified in a general cancer population may therefore not be generalizable to AYA patients.

Within the AYA population, we found that those with hematologic cancers were nearly 25% less likely to be screened with the ESAS tool than those AYA with breast cancer; no other cancer types were similarly disadvantaged. This result is particularly concerning, given findings of substantial symptom burdens among this group of patients.^32^, ^33^, ^34^ Previous authors have called attention to barriers in instituting PROMs for patients with hematologic malignancies, including a lack of disease‐specific validated measures.^35^ It is worth noting, however that ESAS does screen for both nausea and pain, two symptoms commonly experienced by this population. Further efforts to increase symptom screening uptake in this group are warranted.

Adolescents and young adults living in the Northern Ontario, an area traditionally considered disadvantaged, were in fact more likely to be ESAS screened than those in Toronto, the largest urban area in the province. Similarly, AYA living in rural areas were just as likely to be screened as those in the highest income urban neighborhoods. A possible explanation for this surprising finding may relate to the centralization of cancer care in rural and remote areas to a limited number of institutions. Past interventions targeting ESAS screening rates in these institutions may have thus reached patients from across Northern and rural Ontario.^26^ Alternatively, high‐volume cancer centers in urban areas like Toronto may have fewer opportunities to explain the goal and logistics of ESAS screening to individual AYA. Indeed, AYA living in low‐income urban areas were least likely to be ESAS screened. Given near zero correlation in patient characteristics within centers, indicating that the screening rates did not cluster by center, this is unlikely to be due to such AYA attending different cancer centers than higher‐income AYA. Future studies should determine whether low socioeconomic status AYA are less likely to be offered symptom screening, less likely to participate in screening when offered, or both, as such data will be critical in informing the design of future interventions. Though ESAS is available in multiple languages, low literacy in English or other languages may also be a particularly relevant barrier in low socioeconomic status AYA.

One limitation of the ESAS, and of other general PROMs, is that they may not capture all symptoms of most relevance to AYA. For example, AYA have in previous studies noted the importance of changes in appearance, symptoms related to sexual function, or broader impacts on school or relationships.^27^, ^36^ In addition, symptoms specific to the treatment of certain malignancies common in the AYA age range (e.g., tinnitus in patients treated for testicular cancer) are not captured. Whether the use of PROMs specific to malignancies common among AYA, or of malignancy agnostic but AYA‐specific PROMs,^36^, ^37^ would increase uptake among AYA is unknown but merits future study.

Study strengths include its population‐based nature and large sample size. Indeed, this AYA cohort is, to our knowledge, the largest to date for whom symptom assessments were available. Several limitations should also be noted. First, the lack of a completed ESAS screen does not necessarily mean that symptom burden was not assessed and addressed during a clinical encounter. Conversely, a completed ESAS screen does not guarantee interventions to address symptoms. However, multiple studies have shown that PROMs more accurately describe symptom burden in cancer patients as compared to healthcare provider assessments, and lead to improved quality of life.^3^, ^7^, ^8^, ^9^, ^10^, ^11^, ^38^ Second, the generalizability of our finding to other jurisdictions is unknown, particularly ones without universal healthcare insurance where barriers to high quality AYA cancer care may be higher.^39^ Third, our outcome was binary: whether or not AYA were ESAS screened during the first year after cancer diagnosis. ESAS screening would ideally occur at multiple points of the cancer journey, when symptom burdens were expected to change, and after supportive care interventions. Our analyses do not take these factors into account. Fourth, as noted above, while all centers are instructed to offer ESAS screening to all patients at each visit and have infrastructure to enable this, we cannot distinguish whether AYA were not offered screening versus were offered but declined to complete screening. Finally, our study excluded AYA who were diagnosed and treated in pediatric hospitals. Patterns of symptom assessment are likely to differ in pediatric centers given differences in both volume and case mix as compared to adult institutions.

In conclusion, we found that while AYA living in rural and remote areas had high rates of symptom screening in the context of a provincial screening program, AYA with hematologic cancer and those living in low‐income urban areas were substantially less likely to be screened. Additional studies of the mechanisms underlying these disparities are warranted in order to inform the design of future interventions, which may include the introduction of AYA‐specific PROMs.

## CONFLICT OF INTEREST

The authors declare no relevant conflict of interest.

## Supporting information

Supplementary Material

## Data Availability

Privacy legislation in Ontario, Canada prevents the sharing of personal health information.
