# Supplementary figures and images for: The effectiveness of a provincial symptom assessment program in reaching adolescents and young adults with cancer: A population‐based cohort study
Source: Cancer Med. 2021 Nov 5;10(24):9030–9. doi: 10.1002/cam4.4401 (PMC8683532; doi:10.1002/cam4.4401)

Appendix 1


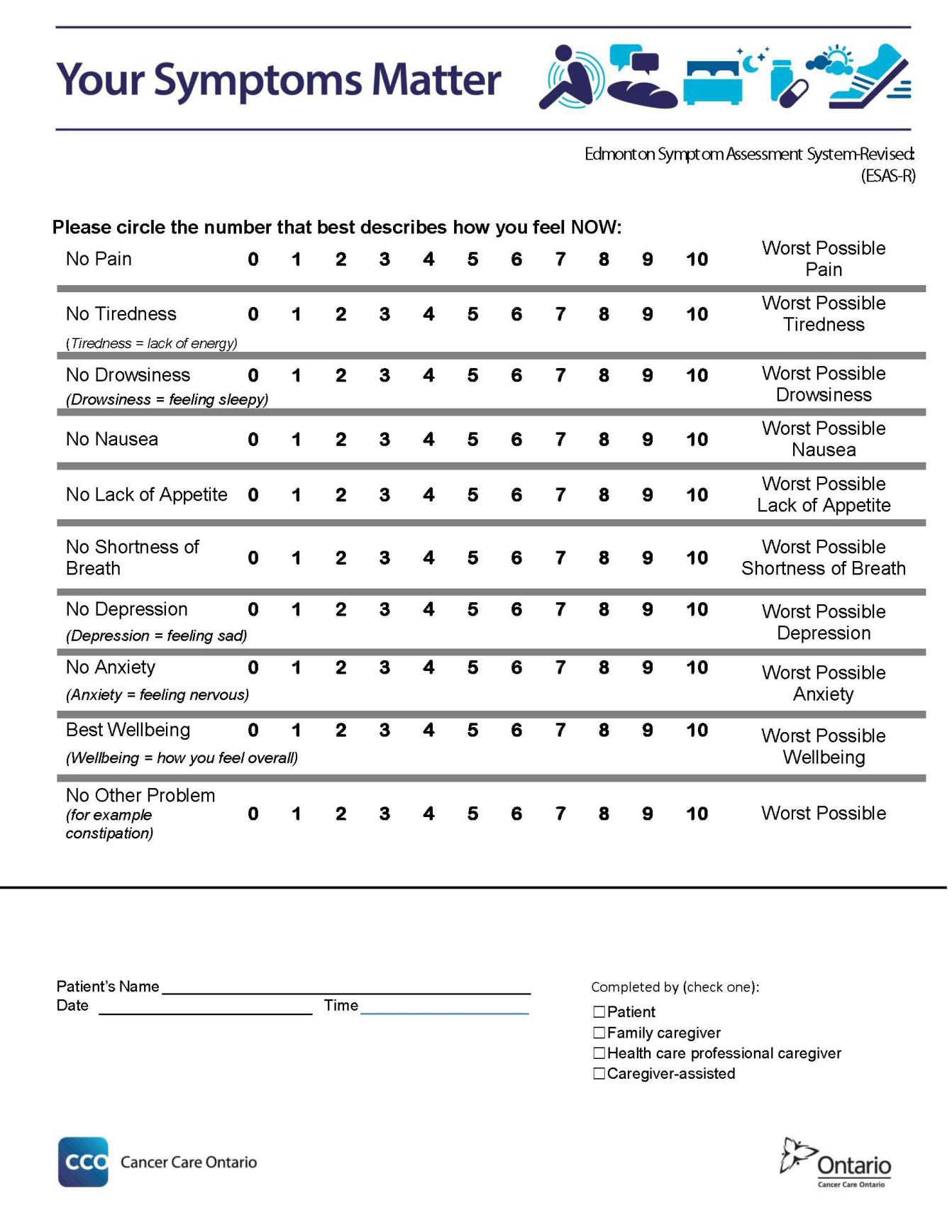

Supplement: Supplementary file 1 — Supplementary Material [file CAM4-10-9030-s001.docx]
